# Tracing inflammasomes in Alzheimer’s: insights from bibliometric analysis

**DOI:** 10.3389/fneur.2025.1540083

**Published:** 2025-06-05

**Authors:** Yingjun Chen, Hui Pei, Hao Li, Wenjun Zeng, Zhitao Li

**Affiliations:** ^1^Graduate School, Beijing University of Chinese Medicine, Beijing, China; ^2^Department of Geriatrics, Xiyuan Hospital, China Academy of Chinese Medical Sciences, Beijing, China; ^3^Wangjing Hospital, China Academy of Chinese Medical Sciences, Beijing, China; ^4^First School of Clinical Medicine, Shandong University of Traditional Chinese Medicine, Jinan, China

**Keywords:** Alzheimer’s disease, inflammasome, bibliometrics, CiteSpace, VOSviewer

## Abstract

**Background:**

Alzheimer’s disease is one common type of dementia. Numerous studies have suggested a correlation between Alzheimer’s disease and inflammation. The inflammasome is the core of the inflammatory response and plays an important role in the inflammatory response. Currently, ample evidence has shown that inflammasomes are closely related to the occurrence and development of Alzheimer’s disease.

**Objective:**

To explore the evolution and development trends of inflammasomes in Alzheimer’s disease using bibliometric and knowledge mapping analysis. By identifying research hotspots and emerging topics, we aim to provide new insights and directions for researchers in this field.

**Methods:**

All data related to inflammasomes in Alzheimer’s disease from 2000 to 2024 were collected from the Web of Science Core Collection (WoSCC), and annual publications, national publication trends, and proportion charts were analyzed and plotted using GraphPad price v8.0.2. Additionally, CiteSpace (6.2.4R (64-bit) Advanced Edition), and VOSviewer (version 1.6.18) were used to analyze and visualize these data.

**Results:**

A total of 1,128 publications related to the inflammasome in Alzheimer’s disease were recorded in the WoSCC, comprising 738 articles and 390 reviews. The literature was mainly from 68 countries/regions and 1,545 institutions, particularly China (*n* = 464) and the USA (*n* = 266). Despite China’s leading in publication quantity, the United States holds a prominent position in the field due to the higher quality of its scholarly articles. The institution that contributes the most publications is the Helmholtz Association. JOURNAL OF MOLECULAR SCIENCES was a prolific contributor, and Nature was the most frequently cited journal. Keyword analysis showed that nlrp3 inflammasome, neuroinflammation, microglia activation, and amyloid-beta were the most common terms, reflecting the main research interests in currently published papers in this field. Research in this field primarily focuses on the NLRP3 inflammasome, which is closely associated with pathological products like Aβ and tau proteins. It can induce pyroptosis and accelerate the progression of Alzheimer’s disease.

**Conclusion:**

The NLRP3 inflammasome is critical in Alzheimer’s disease (AD) pathogenesis. However, the peak of related literature was in 2023, suggesting a potential decline in this research hotspot. There is an urgent need to explore new pathogenic mechanisms for AD. Clearly, this is an important direction that requires deep thinking and breakthroughs.

## 1 Introduction

Alzheimer’s disease is a key factor in cognitive impairment, with its pathogenesis still not fully understood. It is mainly characterized by the deposition of senile plaques made of *β*-amyloid proteins and neurofibrillary tangles formed by abnormal tau protein phosphorylation (1). However, this framework does not completely explain the disease’s etiology. Recently, neuroinflammatory responses have gained attention in Alzheimer’s research, with inflammasome activation considered a critical aspect of the disease’s progression (2).

Inflammasomes are signaling protein complexes that regulate immune cells. They detect exogenous pathogens and endogenous danger signals through pathogen-associated molecular patterns (PAMPs) and damage-associated molecular patterns (DAMPs), triggering inflammatory responses and programmed cell death (3). In the nervous system, inflammasomes are primarily found in microglial cells (4). While they help engage the immune system and maintain internal balance, their dysregulation can contribute to various diseases, including inflammatory ailments, autoimmune disorders, cardiovascular and metabolic issues, microbiome imbalances, cancer, and neurological conditions. In Alzheimer’s disease, Aβ and tau can activate inflammasomes, leading to neuroinflammation and the release of neurotoxic factors, ultimately causing neuronal damage (5).

**Figure 1:**
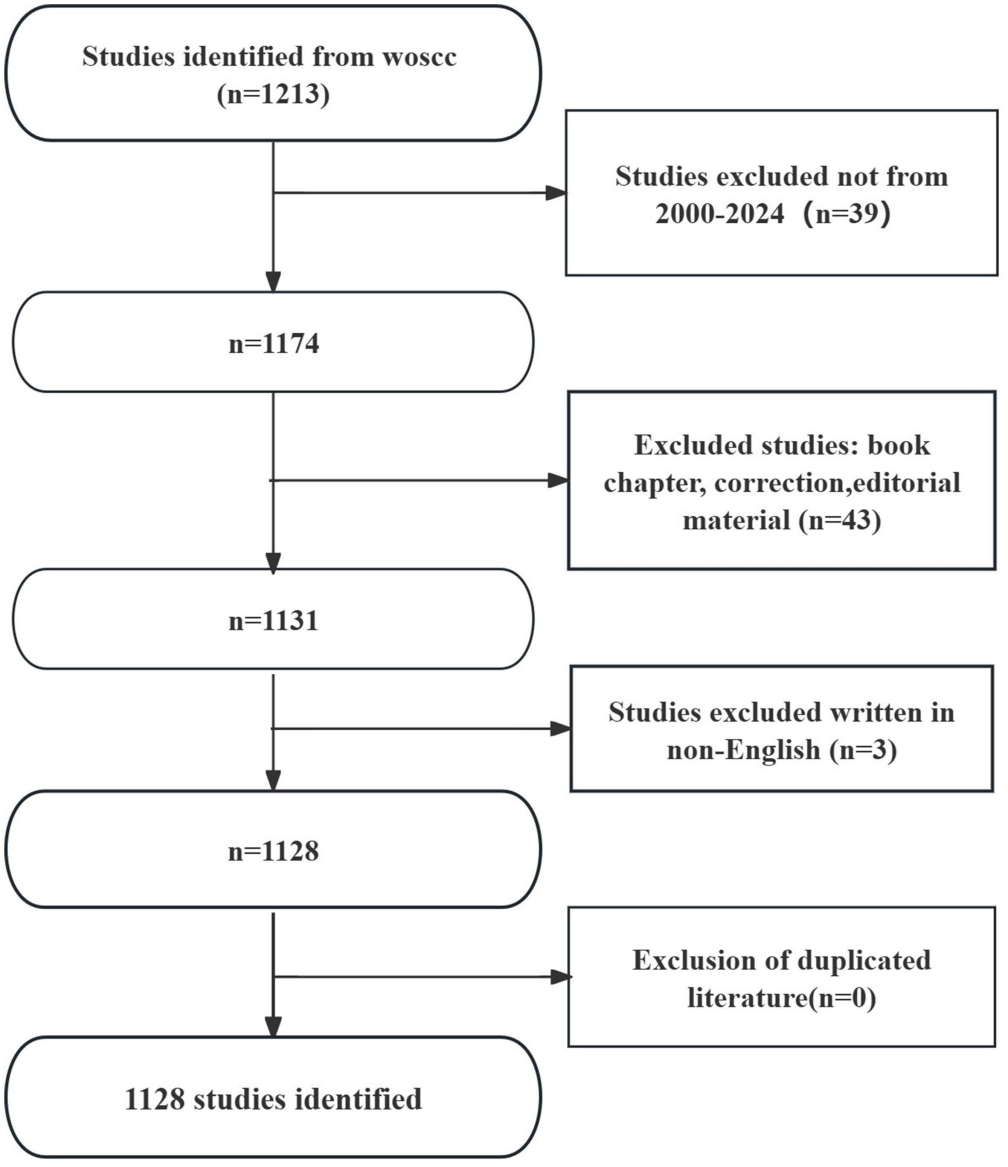
Flowchart of literature search.

Bibliometrics is a multidisciplinary science that uses mathematical and statistical methods to quantitatively analyze knowledge dissemination, such as literature. By thoroughly examining a large body of literature on specific topics, bibliometrics provides valuable insights into: (a) Nations/regions, institutions, and journals involved in the research; (b) Collaborations among countries, institutions, or authors; (c) Journal distribution patterns; (d) Knowledge repositories. Thus, bibliometrics is a valuable tool for researchers to quickly understand a field, identify research focal points, and track evolving trends, helping to avoid redundant studies (Figure 1) (6).

## 2 Methods

### 2.1 Data collection

The Web of Science Core Collection (WoSCC) database is renowned for its superior accuracy in document type labeling, making it the preferred choice for bibliographic analysis. Consequently, we selected this repository for our search (7). On September 16, 2024, we retrieved all articles related to inflammasomes in Alzheimer’s disease research from the year 2000 to September 16, 2024, using the specified search formula. ((((((((((((TS = (Inflammasomes)) OR TS = (Inflammasome)) OR TS = (Pyroptosome)) OR TS = (Pyroptosomes)) OR TS = (NAIP-NLRC4)) OR TS = (NLRP3)) OR TS = (NLRP1)) OR TS = (CARD8)) OR TS = (caspase-1)) OR TS = (caspase-4)) OR TS = (caspase-5)) OR TS = (caspase-11)) OR TS = (GSDMD)) OR TS = (NINJ1) AND ((((((((((((((((((((((((((((TS = (“Alzheimer Disease”)) OR TS = (“Alzheimer Syndrome”)) OR TS = (“Alzheimer-Type Dementia (ATD)”)) OR TS = (“Alzheimer Type Dementia (ATD)”)) OR TS = (“Dementia, Alzheimer-Type (ATD)”)) OR TS = (“Alzheimer’s Diseases”)) OR TS = (“Alzheimer Diseases”)) OR TS = (“Alzheimer Dementia”)) OR TS = (“Alzheimer Dementias”)) OR TS = (“Dementia, Alzheimer”)) OR TS = (“Alzheimer’s Disease”)) OR TS = (“Dementia, Senile”)) OR TS = (“Senile Dementia”)) OR TS = (“Alzheimer Type Dementia”)) OR TS = (“Alzheimer Type Senile Dementia”)) OR TS = (“Primary Senile Degenerative Dementia”)) OR TS = (“Alzheimer Sclerosis”)) OR TS = (“Sclerosis, Alzheimer”)) OR TS = (“Dementia, Primary Senile Degenerative”)) OR TS = (“Presenile Dementia”)) OR TS = (“Acute Confusional Senile Dementia”)) OR TS = (“Senile Dementia, Acute Confusional”)) OR TS = (“Early Onset Alzheimer Disease”)) OR TS = (“Presenile Alzheimer Dementia”)) OR TS = (“Late Onset Alzheimer Disease”)) OR TS = (“Focal Onset Alzheimer’s Disease”)) OR TS = (“Familial Alzheimer Disease (FAD)”)) OR TS = (“Alzheimer Disease, Familial (FAD)”)) OR TS = (“Familial Alzheimer Diseases (FAD)”). The literature selection for this study was guided by the following inclusion criteria: (1) full-text publications related to inflammasomes in Alzheimer’s disease, and (2) articles and review manuscripts written in English. The exclusion criteria included: (1) topics unrelated to inflammasomes in Alzheimer’s disease, and (2) documents such as conference abstracts, news, and briefings. Full-text versions of the selected papers were exported.

### 2.2 Data analysis

Annual publication trends, national publication patterns, and their proportions were analyzed and plotted using GraphPad Prism v8.0.2. Additionally, CiteSpace (version 6.2.4R, 64-bit Advanced Edition) and VOSviewer (version 1.6.18) were employed to analyze the data and visualize the scientific knowledge map.

VOSviewer v1.6.18, developed by Waltman et al. in 2009, is a free Java-based software used for analyzing large volumes of bibliographic data and displaying them in map format (8). To visualize research results in a specific field through literature co-citation network maps, Professor Chao-Min Chen created CiteSpace (version 6.2.4R). This software uses an experimental framework to study new concepts and evaluate existing technologies, enabling users to better understand knowledge domains, research frontiers, and trends, and predict future research progress (9).

## 3 Results

The findings revealed that a total of 1,128 publications pertaining to inflammasome research in Alzheimer’s disease were identified in the WoSCC database, encompassing 738 articles and 390 reviews. This comprehensive analysis involved contributions from 68 countries and regions, as well as collaborations among 1,545 institutions and 5,789 authors.

### 3.1 Annual publication and citation trends

**Figure 2:**
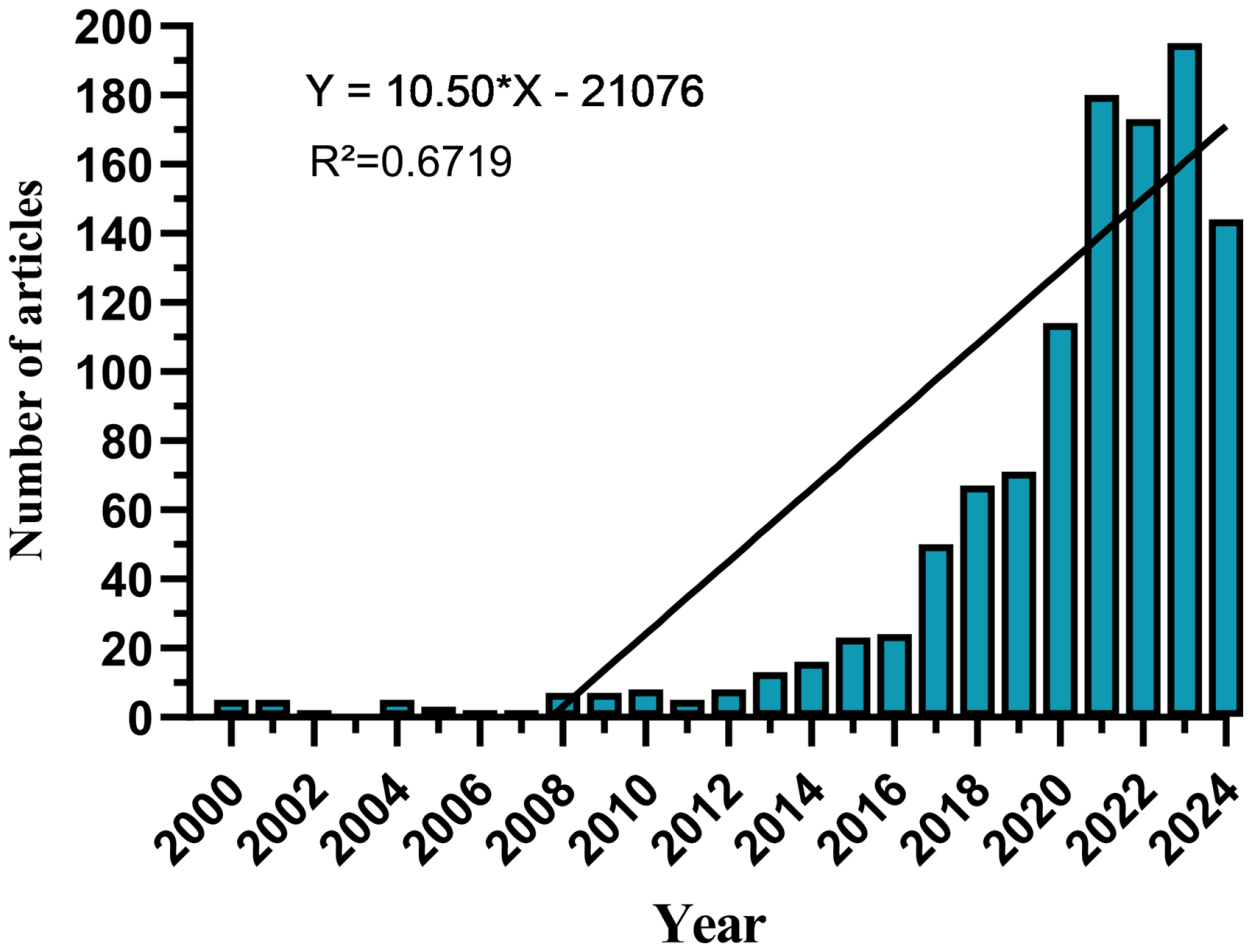
Annual volume of publications.

Since 2000, the annual number of published papers has shown a gradual increase, which can be divided into three stages. From 2000 to 2012, growth was slow (Figure 2), with fewer than 10 papers published annually, suggesting limited development in this field. After 2013, the number of publications rose rapidly, accelerating further after 2020 and peaking in 2023.

### 3.2 Countries and institutions

**Figure 3:**
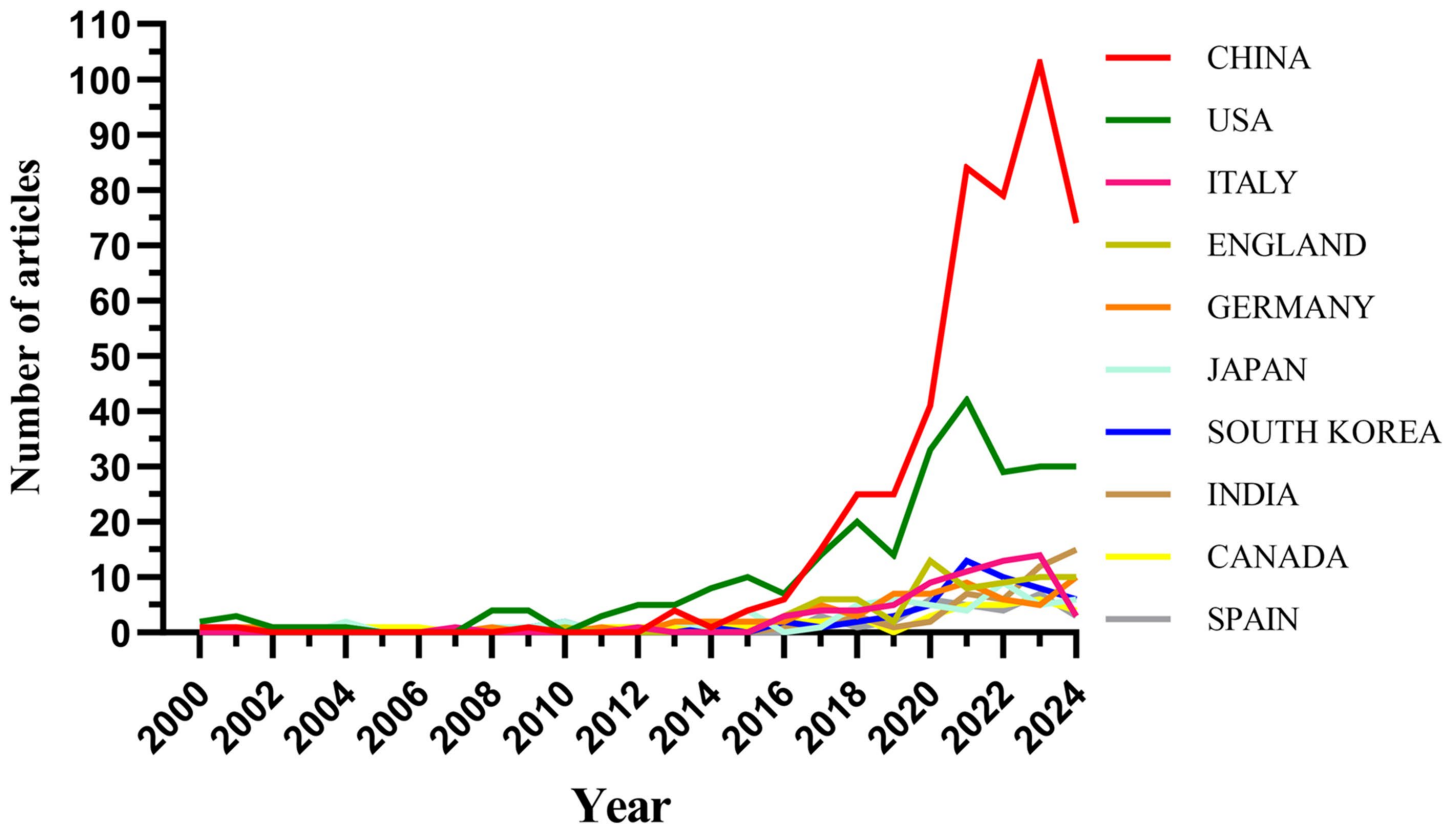
Line graph of national publications.

**Figure 4:**
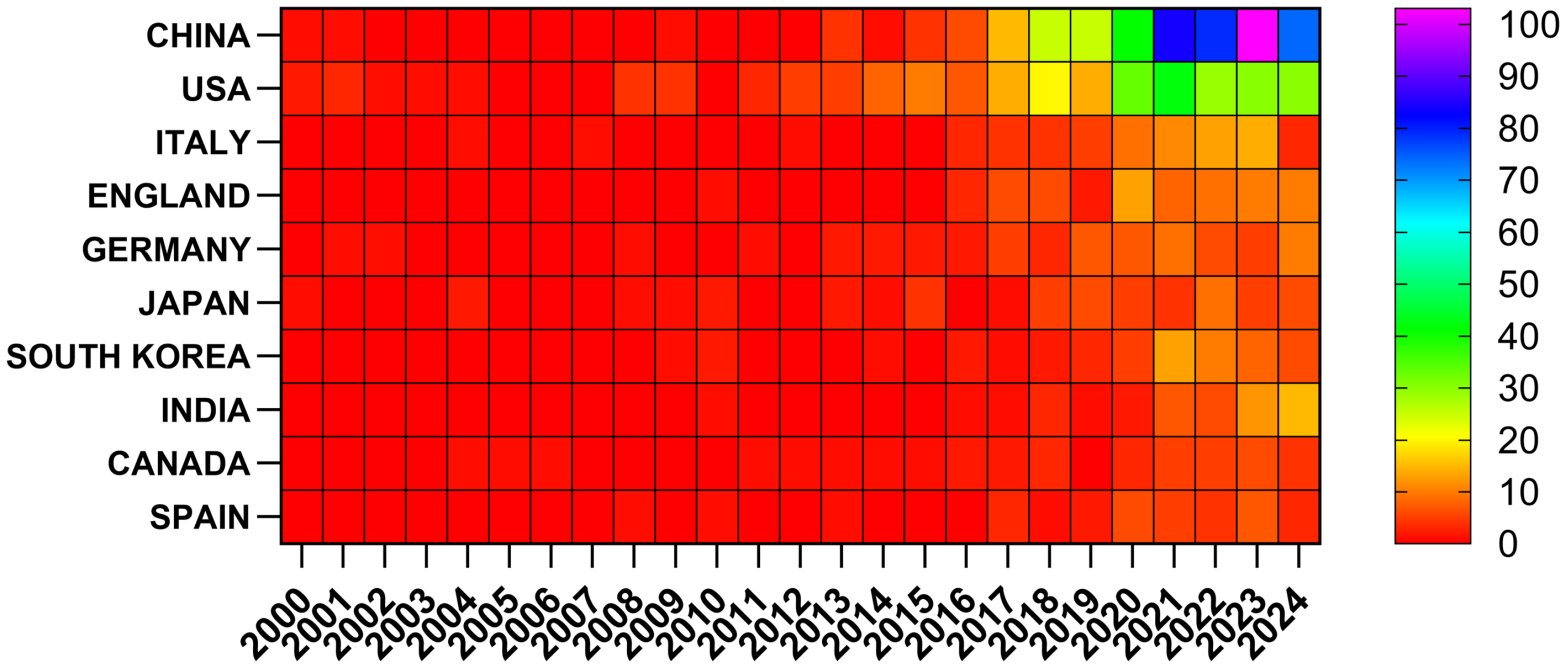
Heat map of national publications.

Research on the related applications of inflammasomes in Alzheimer’s disease has been carried out in 68 countries and regions. Figures 3, 4 show the annual number of published papers in the top 10 countries over the past decade. The top 5 countries in this field are China, the United States, Italy, the United Kingdom, and Germany, respectively. The number of papers published in China accounts for 41.13% of the total number of published papers, far exceeding that of other countries.

**Figure 5:**
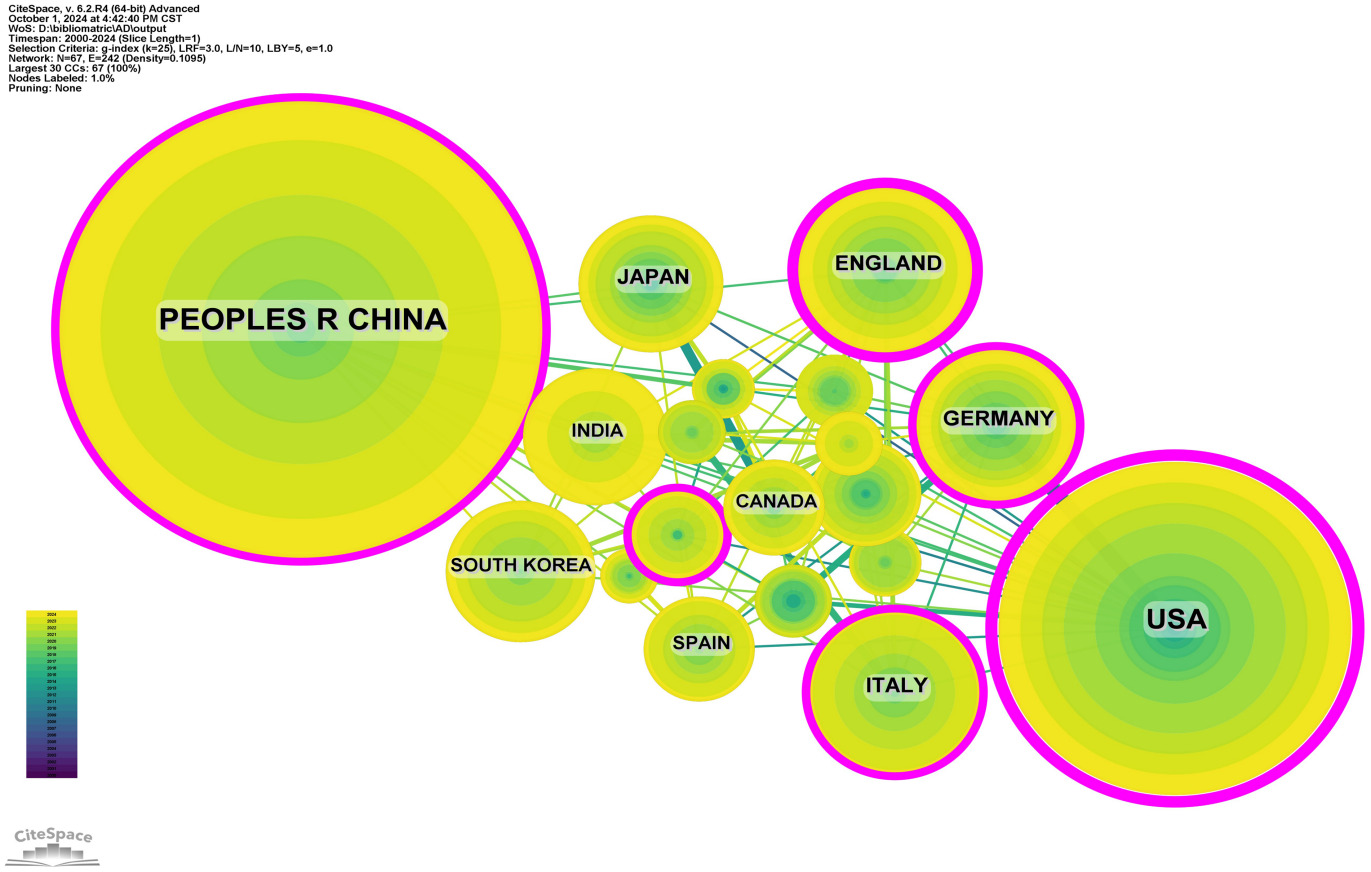
Networks of country cooperation.

Among the top ten countries/regions in terms of the number of published papers, the United States leads with 24,534 citations (Table 1), far surpassing other countries. Its citation-to-publication ratio of 92.23 ranks third globally, indicating a high overall quality of its publications. China, with the highest number of publications (464 papers), ranks first in quantity and second in citations (13,975), but its citation-to-publication ratio of 30.12 suggests a generally lower quality. The cooperation network is illustrated in Figure 5. The United States collaborates closely with the United Kingdom, Italy, and Germany, while China partners more with Japan, South Korea, and India. Despite this, China’s centrality score of 0.13 signifies its prominent role in the field.

**Table 1:** Table of country published literature.

Rank	Country/region	Article counts	centrality	Percentage (%)	Citation	Citation per publication
1	CHINA	464	0.13	41.13%	13,975	30.12
2	USA	266	0.54	23.58%	24,534	92.23
3	ITALY	69	0.15	6.12%	3,844	55.71
4	ENGLAND	68	0.27	6.03%	3,182	46.79
5	GERMANY	64	0.2	5.67%	11,314	176.78
6	JAPAN	55	0.06	4.88%	2,803	50.96
7	SOUTH KOREA	54	0.01	4.79%	1973	36.54
8	INDIA	49	0.08	4.34%	1,160	23.67
9	CANADA	38	0.05	3.37%	2,256	59.37
10	SPAIN	34	0.05	3.01%	3,502	103.00

### 3.3 Institutions of paper publication

**Figure 6:**
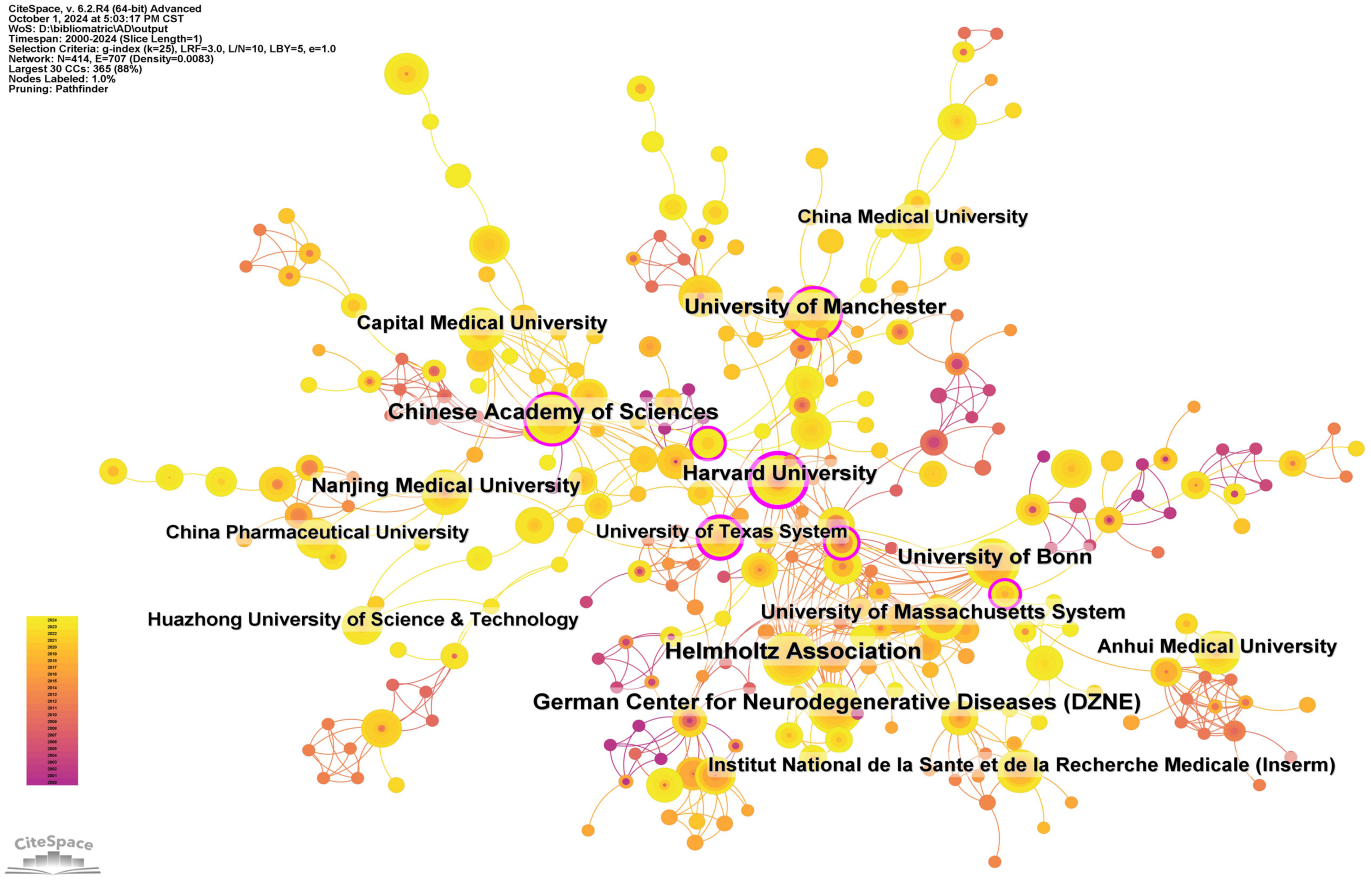
Networks of Institutional Co-operation.

**Figure 7:**
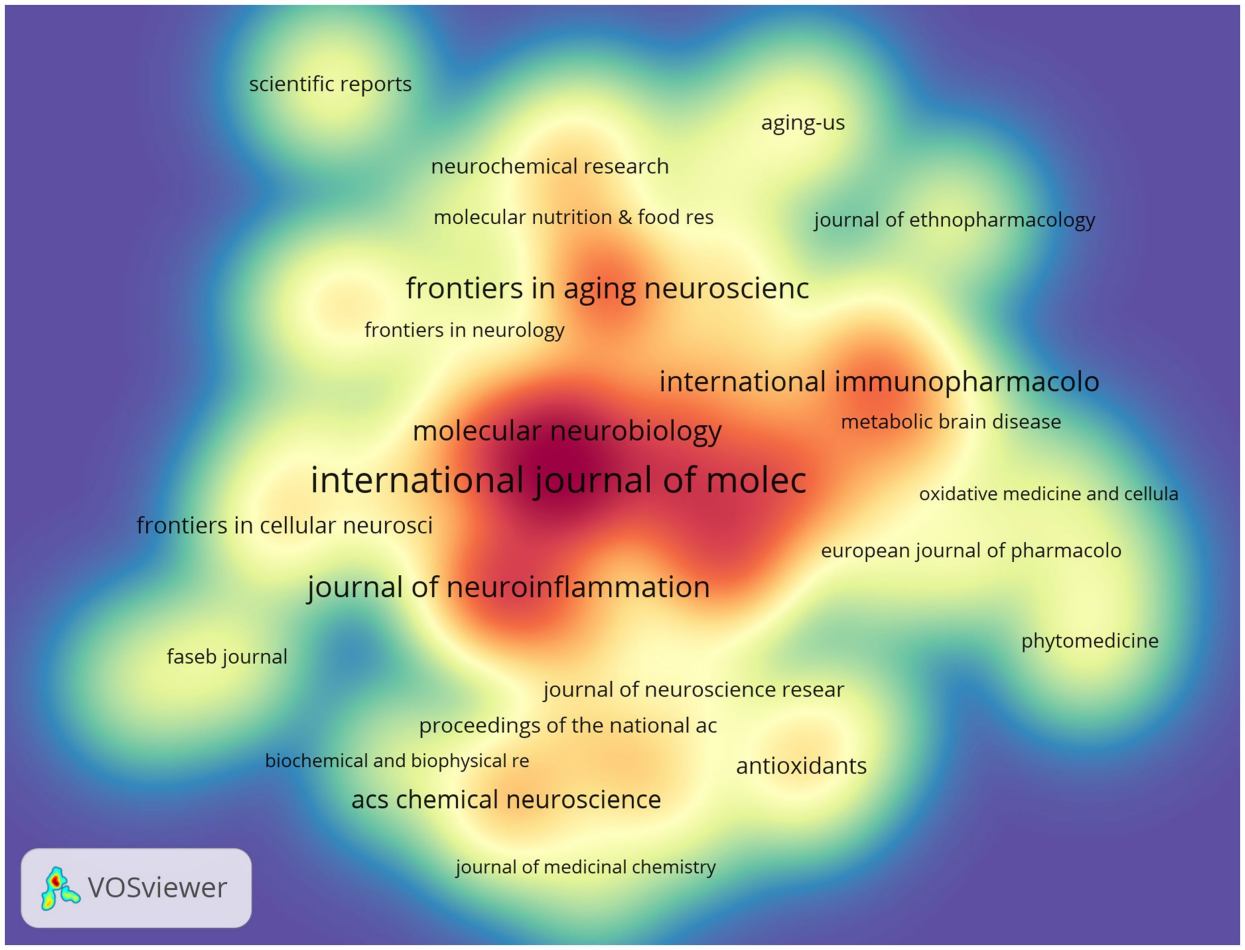
Density map of journal publications.

A total of 1,545 institutions have systematically published articles on inflammasomes in Alzheimer’s disease (Table 2 and Figure 6). Among the top ten institutions by publication volume, four are from China, three from Germany, two from the United Kingdom, and one from the United States. The Helmholtz Association leads with 28 papers and 7,640 citations, averaging 272.86 citations per paper. The German Center for Neurodegenerative Diseases (DZNE) follows with 23 papers and 7,270 citations, averaging 316.09 citations per paper. The Chinese Academy of Sciences ranks third with 22 papers and 1,285 citations, averaging 58.41 citations per paper, while Harvard University ranks fourth with 20 papers and 3,367 citations, averaging 168.35 citations per paper (Figure 7).

**Table 2:** Table of institutional published literature.

Rank	Institution	Country	Number of studies	Total citations	Average citation
1	Helmholtz Association	Germany	28	7,640	272.86
2	German Center for Neurodegenerative Diseases (DZNE)	Germany	23	7,270	316.09
3	Chinese Academy of Sciences	China	22	1,285	58.41
4	Harvard University	USA	20	3,367	168.35
5	University of Bonn	Germany	20	5,038	251.90
6	University of Manchester	England	19	970	51.05
7	University of Massachusetts System	England	17	6,625	389.71
8	Nanjing Medical University	China	17	940	55.29
9	Capital Medical University	China	16	451	28.19
10	Anhui Medical University	China	16	351	21.94

### 3.4 Journals for paper publication

Tables 3, 4 highlight the top 10 journals by output and citations in the field. The *International Journal of Molecular Sciences* leads with 50 papers (4.43%), followed by the *Journal of Alzheimer’s Disease* and the *Journal of Neuroinflammation*, each with 31 papers (2.75%), and *Frontiers in Immunology* with 30 papers (2.66%). Among these, the *Journal of Neuroinflammation* has the highest Impact Factor (IF) of 9.3. All these journals are classified as Q1/Q2.

**Table 3:** Table of journal publications.

Rank	Journal	Article counts	Percentage(1128)	IF	Quartile in category
1	International journal of molecular sciences	50	4.43%	4.9	Q1
2	Journal of Alzheimer’s disease	31	2.75%	3.4	Q2
3	Journal of neuroinflammation	31	2.75%	9.3	Q1
4	Frontiers in immunology	30	2.66%	5.7	Q1
5	Frontiers in aging neuroscience	29	2.57%	4.1	Q2
6	Molecular neurobiology	25	2.22%	4.6	Q1
7	Cells	24	2.13%	5.1	Q2
8	International immunopharmacology	24	2.13%	4.8	Q1
9	Frontiers in pharmacology	23	2.04%	4.4	Q1
10	acs chemical neuroscience	17	1.51%	4.1	Q2

**Table 4:** Co-citation table of journals.

Rank	Cited Journal	Co-Citation	IF(2023)	Quartile in category
1	NATURE	927	50.5	Q1
2	P NATL ACAD SCI USA	719	9.4	Q1
3	J BIOL CHEM	671	4.0	Q2
4	CELL	654	45.5	Q1
5	J NEUROINFLAMM	642	9.3	Q1
6	J NEUROSCI	641	4.4	Q1
7	PLOS ONE	618	2.9	Q1
8	J ALZHEIMERS DIS	617	3.4	Q2
9	SCIENCE	613	44.7	Q1
10	NAT IMMUNOL	592	27.7	Q1

**Figure 8:**
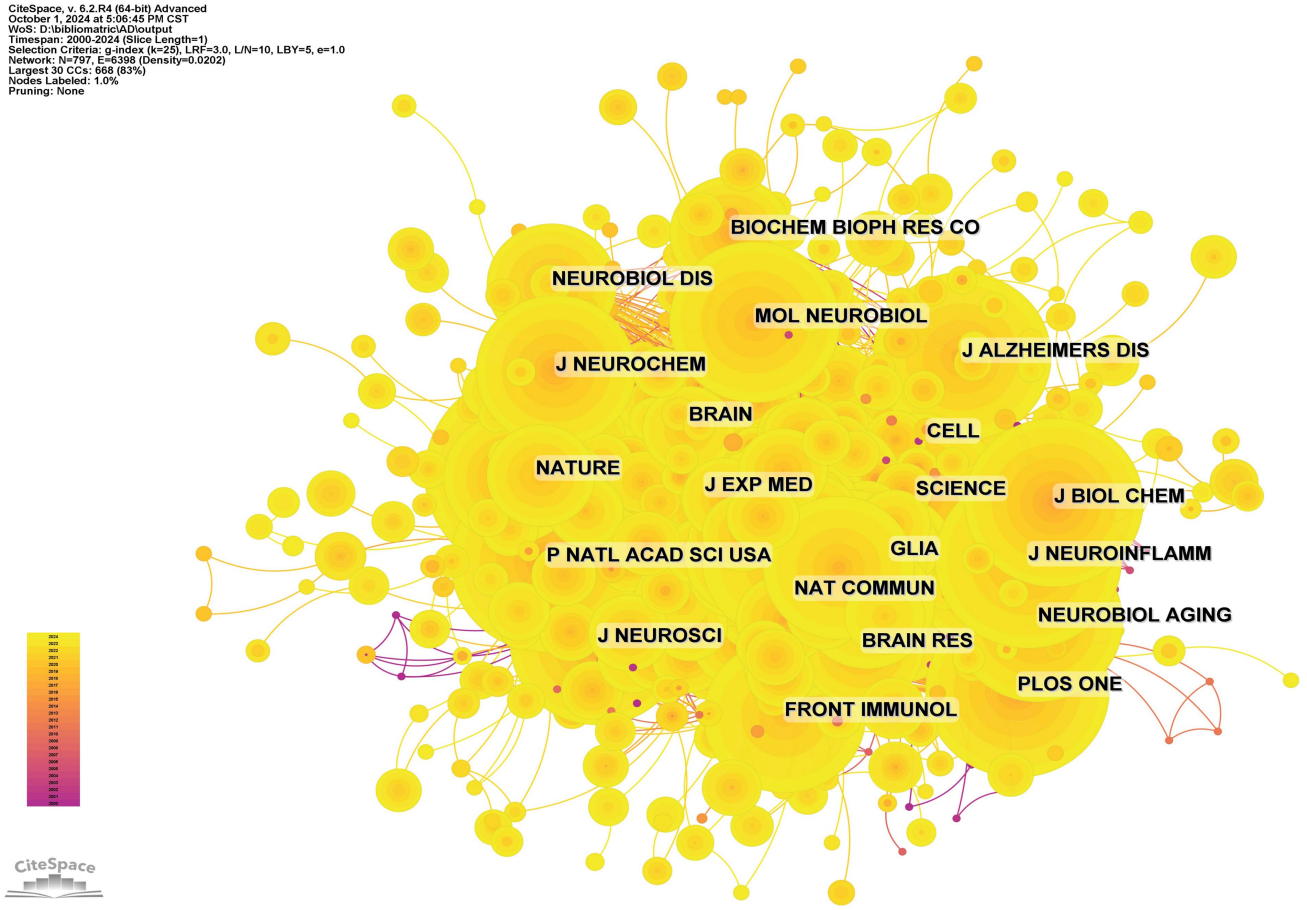
Co-citation network map of journals.

**Figure 9:**
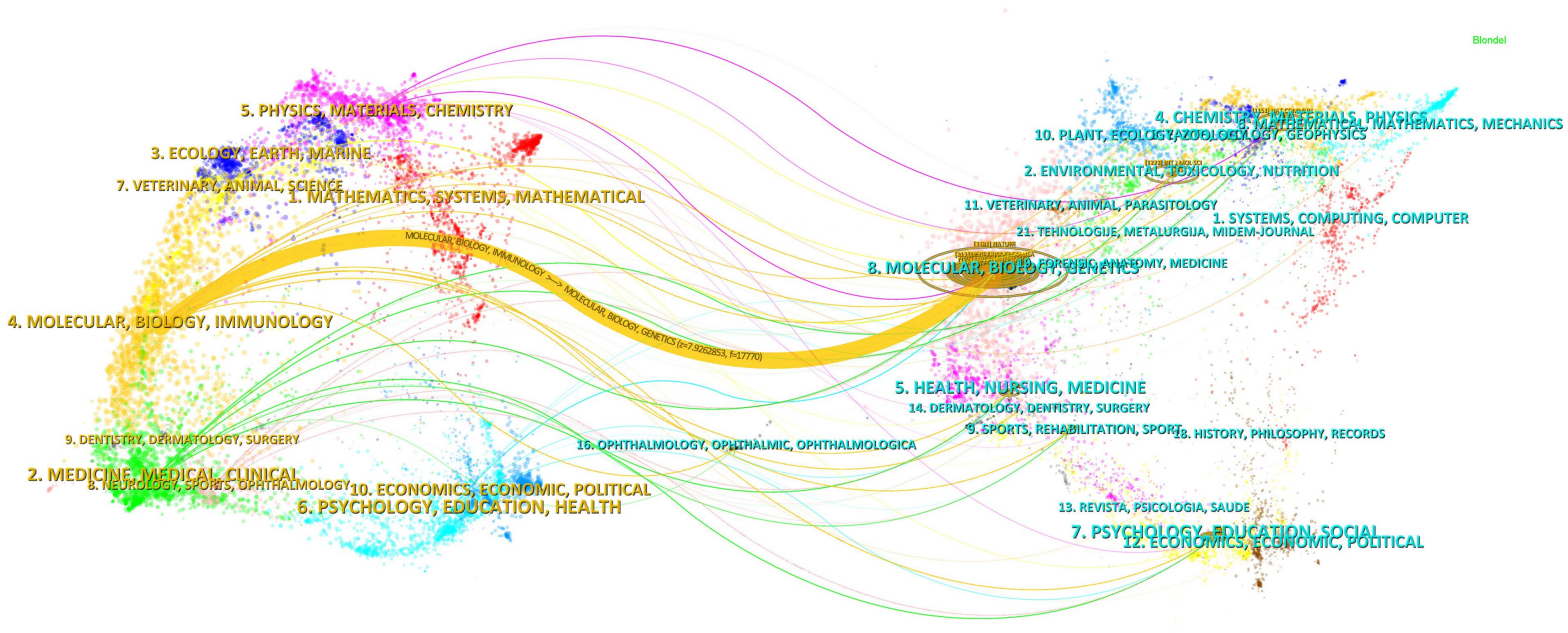
Dual map of journals.

The influence of a journal is often determined by its co-citation frequency, reflecting its impact on the scientific community. According to Figure 8 and Table 4, *Nature* is the most co-cited journal with 927 citations and has the highest Impact Factor (IF) of 50.5 among the top 10. It is followed by *Proceedings of the National Academy of Sciences of the USA* (719 times) and *Journal of Biological Chemistry* (671 times). All the top co-cited journals are classified in the Q1/Q2 category (Figure 9).

The thematic distribution of academic publications is illustrated using a dual-map overlay, where colored tracks indicate citation links. Citing journals are on the left, and cited journals are on the right. The major citation path identified shows that research published in the molecular/biology/genetics field is primarily cited by research in the molecular/biology/immunology field.

### 3.5 Authors of published papers and co-cited authors

**Figure 10:**
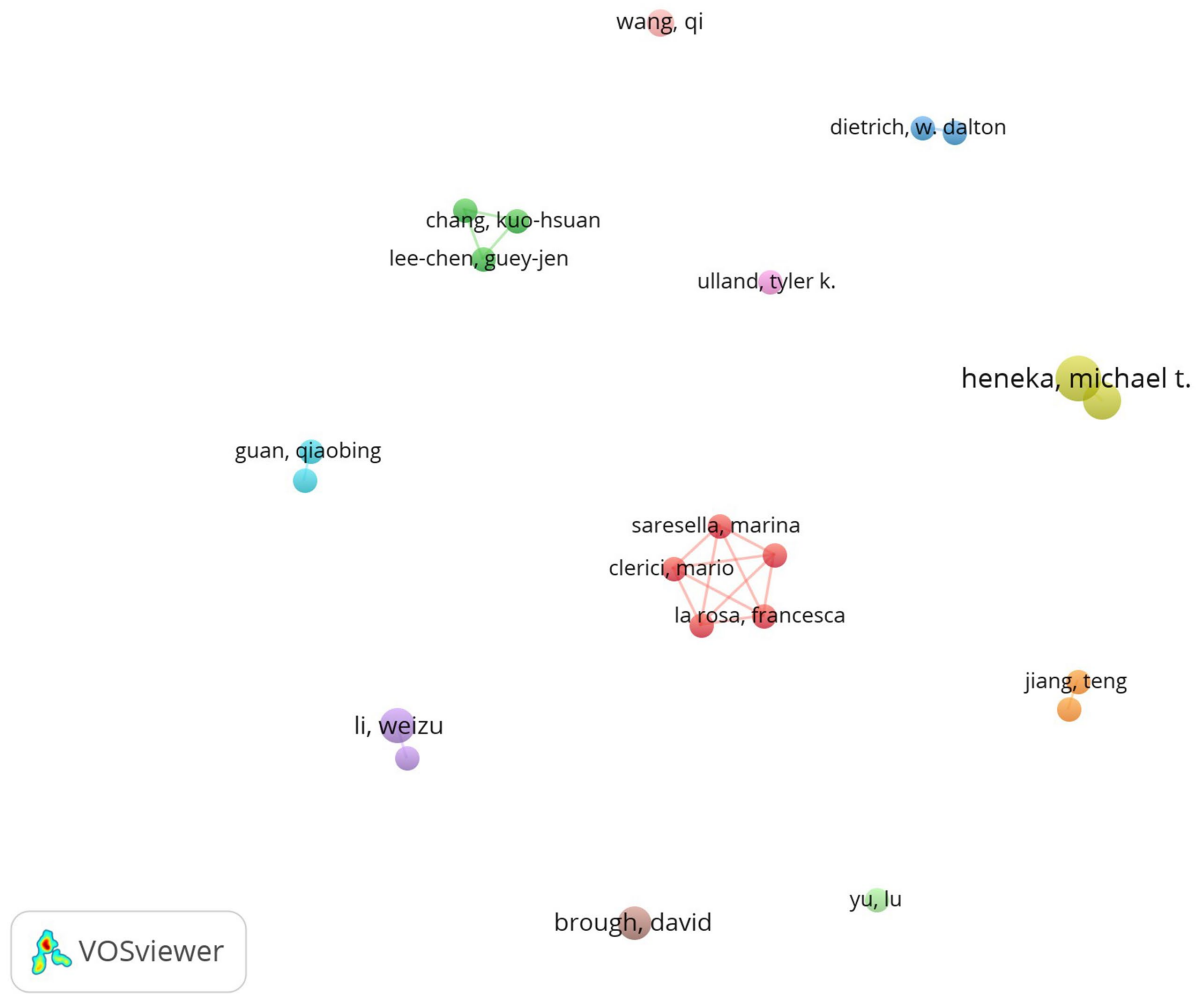
Cooperation network of authors.

Table 5 highlights the top 10 authors with the most publications on inflammasomes in Alzheimer’s disease, contributing a total of 98 papers, which constitutes 8.69% of the field’s literature. The leading author is Heneka, Michael T., with 18 papers, followed by Latz, Eicke (14 papers), Li, Weizu (12 papers), and Brough, David (11 papers). The collaboration network among these authors is visualized using CiteSpace in Figure 10.

**Table 5:** Author’s publications and co-citation table.

Rank	Author	Count	Rank	Co-cited author	Citation
1	Heneka, michael t.	18	1	Heneka mt	600
2	Latz, eicke	14	2	Halle a	333
3	Li, weizu	12	3	Ising c	239
4	Brough, david	11	4	Venegas c	185
5	Wang, qi	8	5	Martinon f	175
6	Chang, kuo-hsuan	7	6	Tan ms	163
7	Chen, chiung-mei	7	7	Zhou rb	155
8	Clerici, mario	7	8	Coll rc	145
9	Dietrich, w. Dalton	7	9	Dempsey c	143
10	Guan, qiaobing	7	10	Saresella m	140

**Figure 11:**
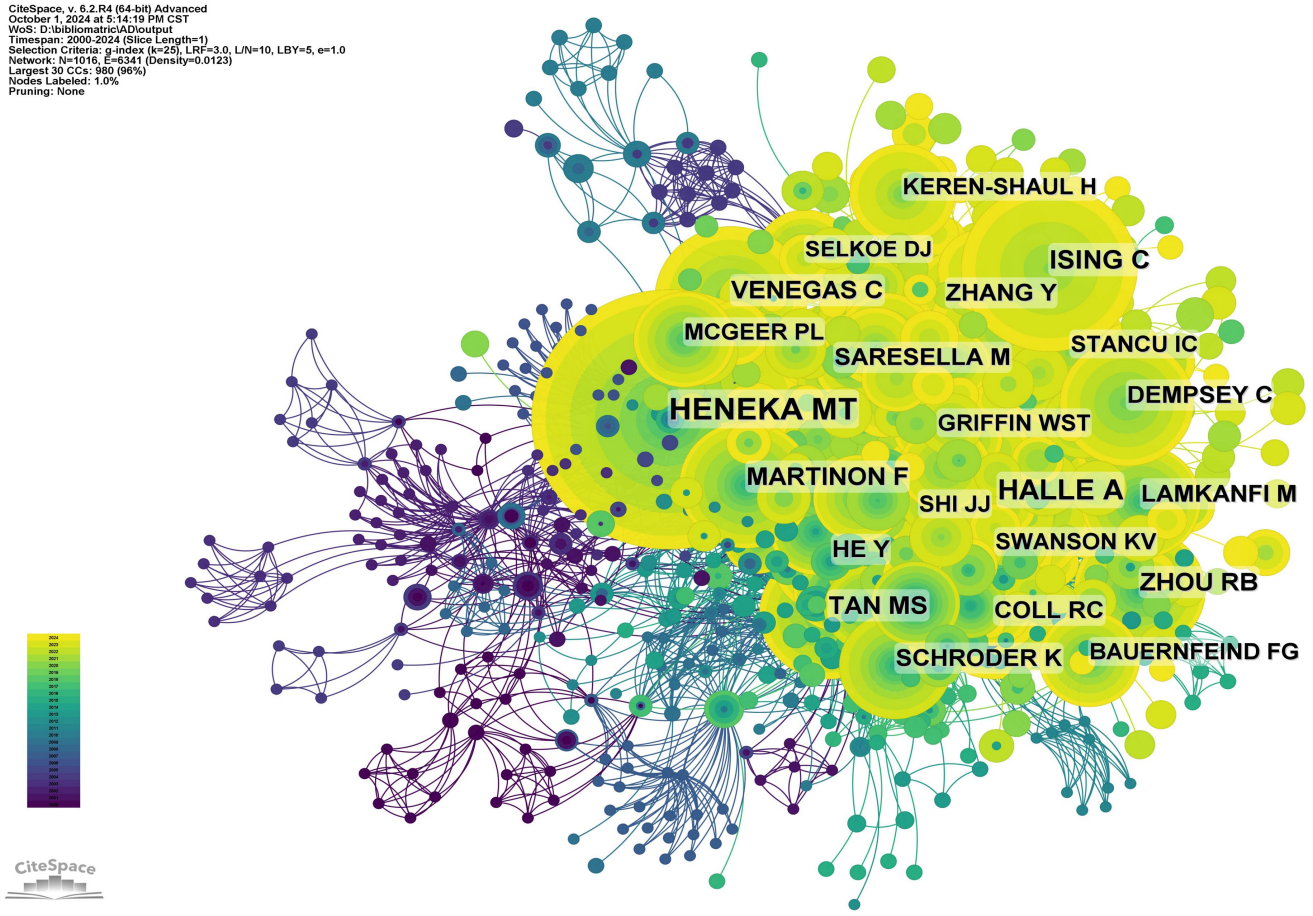
Co-citation network of authors.

Figure 11 and Table 5 present the top 10 most co-cited and most cited authors in the field. A total of 81 authors have been cited over 50 times, underscoring their substantial influence. The largest nodes in the co-citation network are Heneka, Michael T. (600 citations), Halle, A. (333 citations), and Ising, C. (239 citations), highlighting their prominent roles in advancing research on inflammasomes in Alzheimer’s disease.

### 3.6 Co-cited references

**Figure 12:**
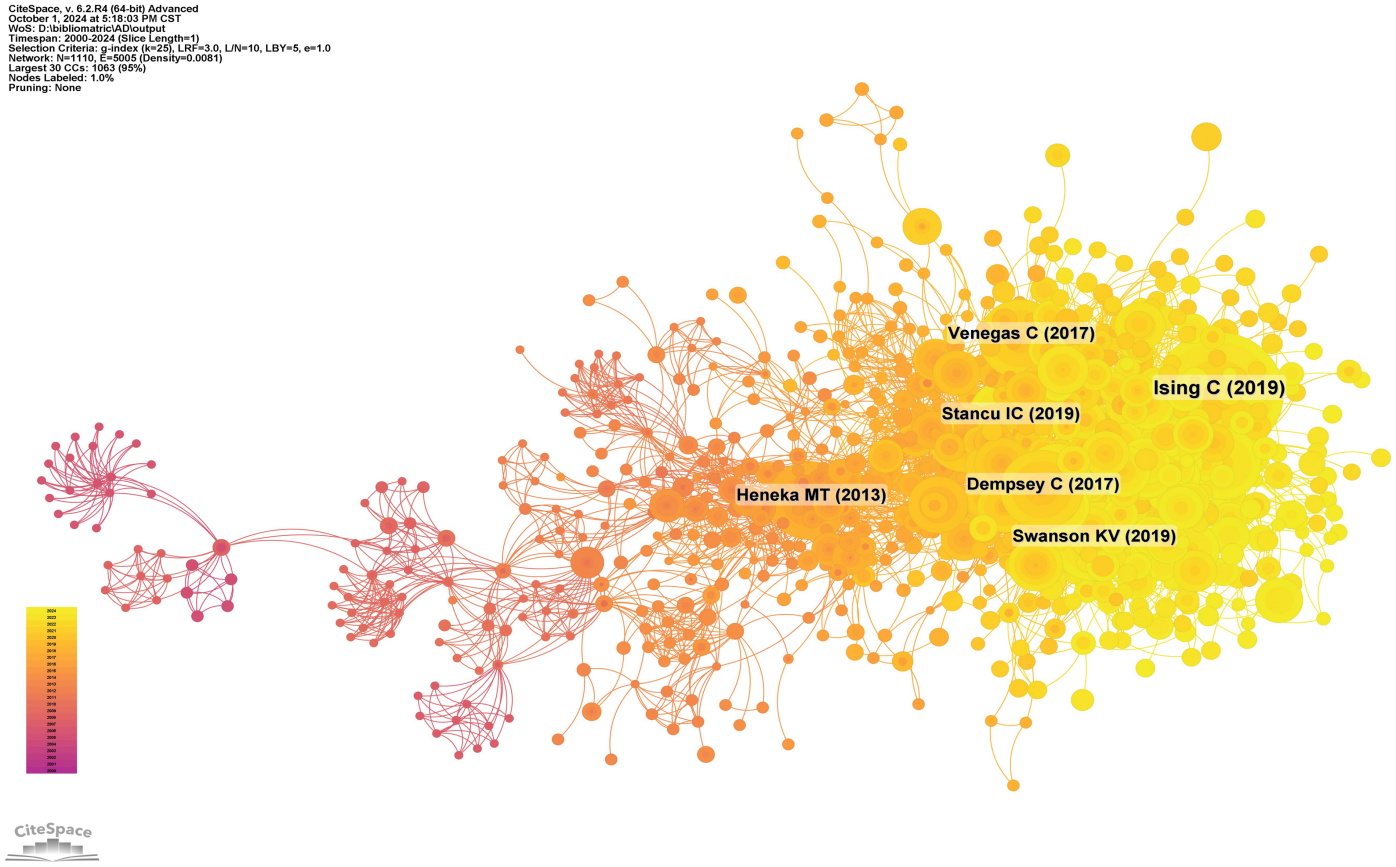
co-cited network of literature.

The co-cited reference network, illustrated in Figure 12, comprises 1,110 nodes and 5,005 links, spanning from 2000 to 2024 with a one-year time slice. Among the top 10 most co-cited articles (Table 6), the article “NLRP3 Inflammasome Activation Drives Tau Pathology,” published in *Nature* with Christina Ising as the first author, is significant.

**Table 6:** Co-citation table of literature.

Rank	Title	Journal	Author(s)	Total citations
1	NLRP3 inflammasome activation drives tau pathology	*NATURE*	Ising C	236
2	Aggregated Tau activates NLRP3-ASC inflammasome exacerbating exogenously seeded and non-exogenously seeded Tau pathology in vivo	*ACTA NEUROPATHOLOGICA*	Stancu IC	105
3	Inhibiting the NLRP3 inflammasome with MCC950 promotes non-phlogistic clearance of amyloid-β and cognitive function in APP/PS1 mice	*BRAIN BEHAVIOR AND IMMUNITY*	Dempsey C	101
4	Microglia-derived ASC specks cross-seed amyloid-β in Alzheimer’s disease	*NATURE*	Venegas C	101
5	The NLRP3 inflammasome: molecular activation and regulation to therapeutics	*NATURE REVIEWS IMMUNOLOGY*	Swanson KV	98
6	NLRP3 is activated in Alzheimer’s disease and contributes to pathology in APP/PS1 mice	*NATURE*	Heneka MT	98
7	The NLRP3 Inflammasome: An Overview of Mechanisms of Activation and Regulation	*INTERNATIONAL JOURNAL OF MOLECULAR SCIENCES*	Kelley N	85
8	Neuroinflammation and microglial activation in Alzheimer disease: where do we go from here?	*NATURE REVIEWS NEUROLOGY*	Leng FD	82
9	The NLRP3 and NLRP1 inflammasomes are activated in Alzheimer’s disease	*MOLECULAR NEURODEGENERATION*	Saresella M	73
10	Inflammasome signaling in brain function and neurodegenerative disease	*NATURE REVIEWS NEUROSCIENCE*	Heneka MT	65

This study suggests that Alzheimer’s disease is characterized by the aggregation of amyloid-*β* in plaques, hyperphosphorylated tau in neurofibrillary tangles, and neuroinflammation, all of which contribute to neurodegeneration and cognitive decline. The NLRP3 inflammasome assembles in microglia upon activation, leading to the cleavage and increased activity of caspase-1 and the subsequent release of interleukin-1*β*.

While the NLRP3 inflammasome is known to be crucial for the development and progression of amyloid-β pathology in mice, its precise impact on tau pathology was unclear. The study found that a deficiency in NLRP3 inflammasome function reduced tau hyperphosphorylation and aggregation by modulating tau kinases and phosphatases. Tau was shown to activate the NLRP3 inflammasome and intracerebral injection of brain homogenates containing fibrillar amyloid-β induced tau pathological changes in an NLRP3-dependent manner. These findings underscore the significant role of microglia and NLRP3 inflammasome activation in the pathogenesis of tauopathy and support the amyloid cascade hypothesis in Alzheimer’s disease, demonstrating that neurofibrillary tangles develop downstream of amyloid-*β*-induced microglial activation (10).

The second-ranked article, “Aggregated Tau Activates NLRP3-ASC Inflammasome Exacerbating Exogenously Seeded and Non-Exogenously Seeded Tau Pathology *In Vivo*,” by Ilie-Cosmin Stancu, published in *Acta Neuropathologica*, explores the interplay between amyloid plaques, neurofibrillary tangles, and neuroinflammation in Alzheimer’s disease. The study highlights the pivotal role of the NLRP3-ASC inflammasome—comprising the NACHT, LRR, and PYD domains-containing protein 3 (NLRP3) and ASC (CARD-containing apoptotic speck-like protein)—in amyloid-β-induced microgliopathy and amyloid pathology.

Previous research established that amyloid-β aggregates activate the NLRP3-ASC inflammasome (Halle et al., *Nat Immunol*, 2008), and that this activation can worsen amyloid pathology *in vivo* (Heneka et al., *Nature*, 2013), including through prion-like ASC speck cross-seeding (Venegas et al., *Nature*, 2017). However, the connection between inflammasome activation and Tau remained unexplored.

This study investigates whether Tau aggregates, acting as prion-like seeds, can activate the NLRP3-ASC inflammasome. The findings reveal that Tau seeds activate the inflammasome in primary microglia following microglial uptake and lysosomal sorting. Furthermore, the study shows that ASC deficiency significantly inhibits exogenously seeded Tau pathology in Tau transgenic mice. Long-term intracerebral administration of the NLRP3 inhibitor MCC950 also reduced exogenous Tau pathology. Additionally, ASC deficiency decreased non-exogenous Tau pathological changes in these mice.

In conclusion, the research demonstrates that aggregated Tau with seeding ability activates the ASC inflammasome via the NLRP3-ASC axis, exacerbating both exogenously and non-exogenously seeded Tau pathology *in vivo*. The NLRP3-ASC inflammasome, a critical sensor of innate immunity, presents a promising therapeutic target for addressing key pathogenic processes in Alzheimer’s disease, including prion-like Tau pathology seeding, amyloid pathology, and neuroinflammation (11).

### 3.7 Co-citation literature and keyword clustering analysis

**Figure 13:**
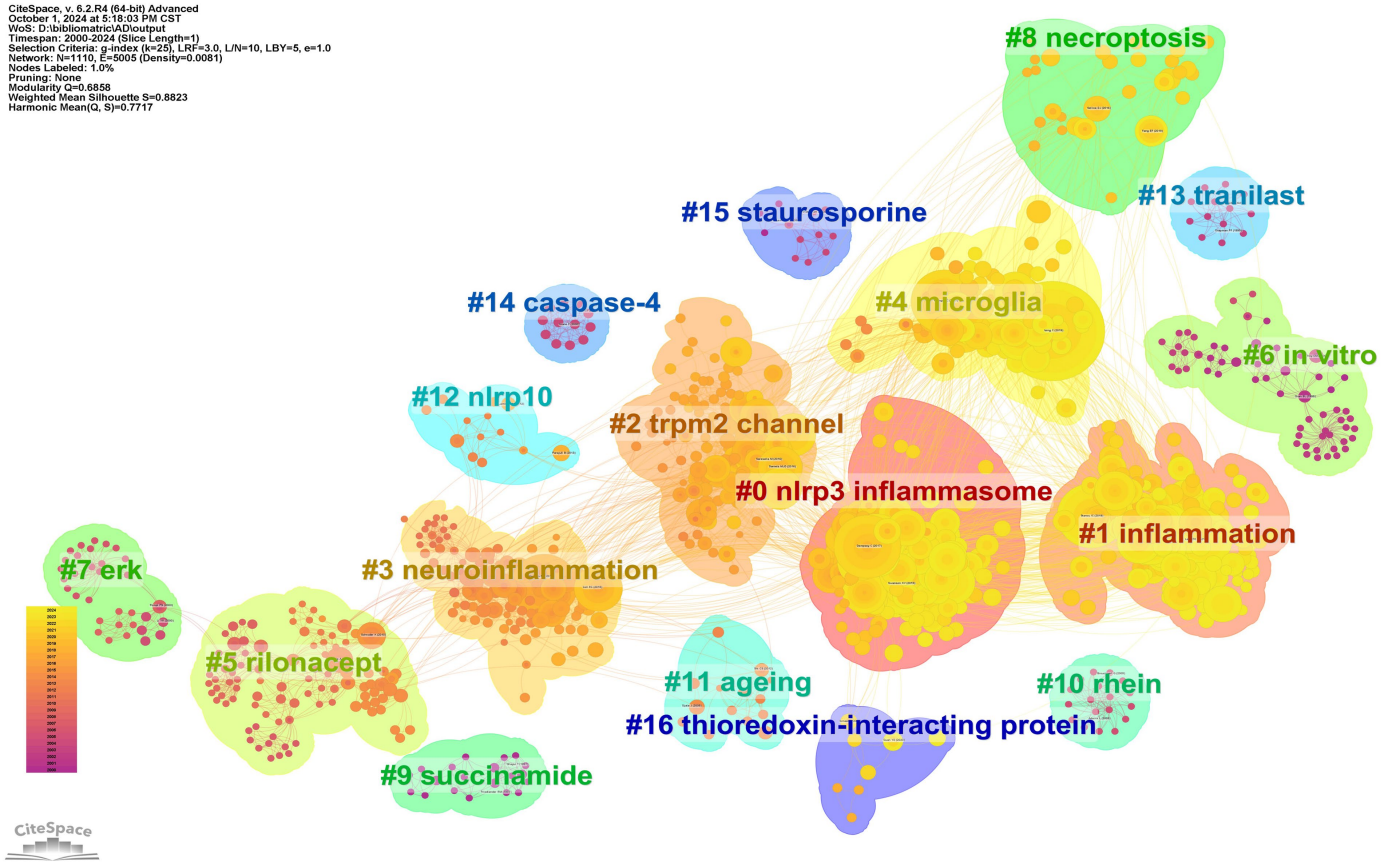
Clustering of co-cited literature.

**Figure 14:**
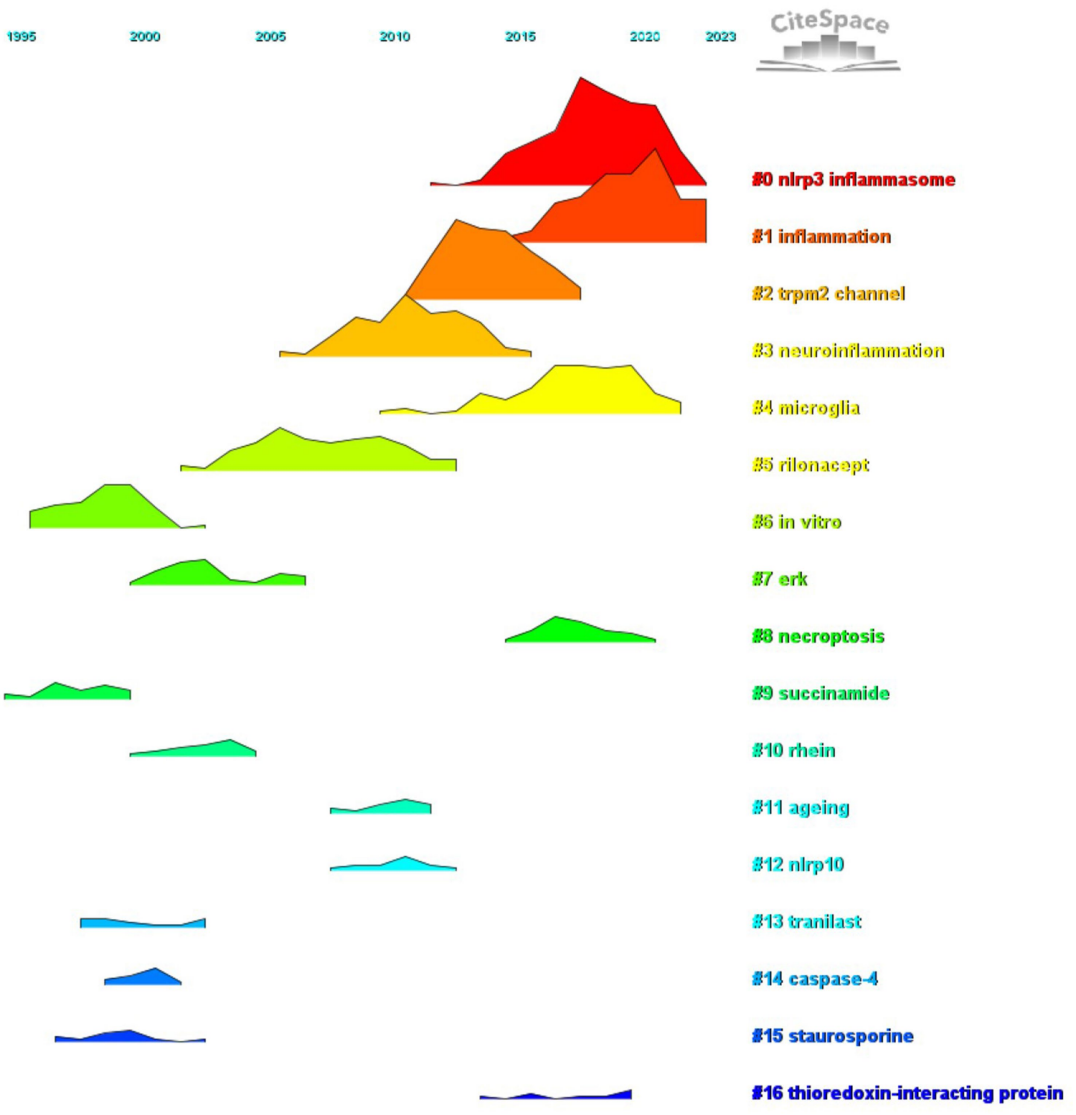
Peak map of co-cited literature.

We conducted co-citation reference clustering and temporal clustering analyses (Figures 13, 14). Our findings indicate that early-stage research hotspots included *in vitro* studies (cluster 6), succinamide (cluster 9), tranilast (cluster 13), caspase-4 (cluster 14), and staurosporine (cluster 15). In the mid-term research phase, key areas of focus were the TRPM2 channel (cluster 2), neuroinflammation (cluster 3), rilonacept (cluster 6), ERK (cluster 7), rhein (cluster 10), aging (cluster 11), and NLRP10 (cluster 13). Currently, the prominent topics and trends in the field are the NLRP3 inflammasome (cluster 0), inflammation (cluster 1), microglia (cluster 4), necroptosis (cluster 8), and thioredoxin-interacting protein (cluster 16).

**Figure 15:**
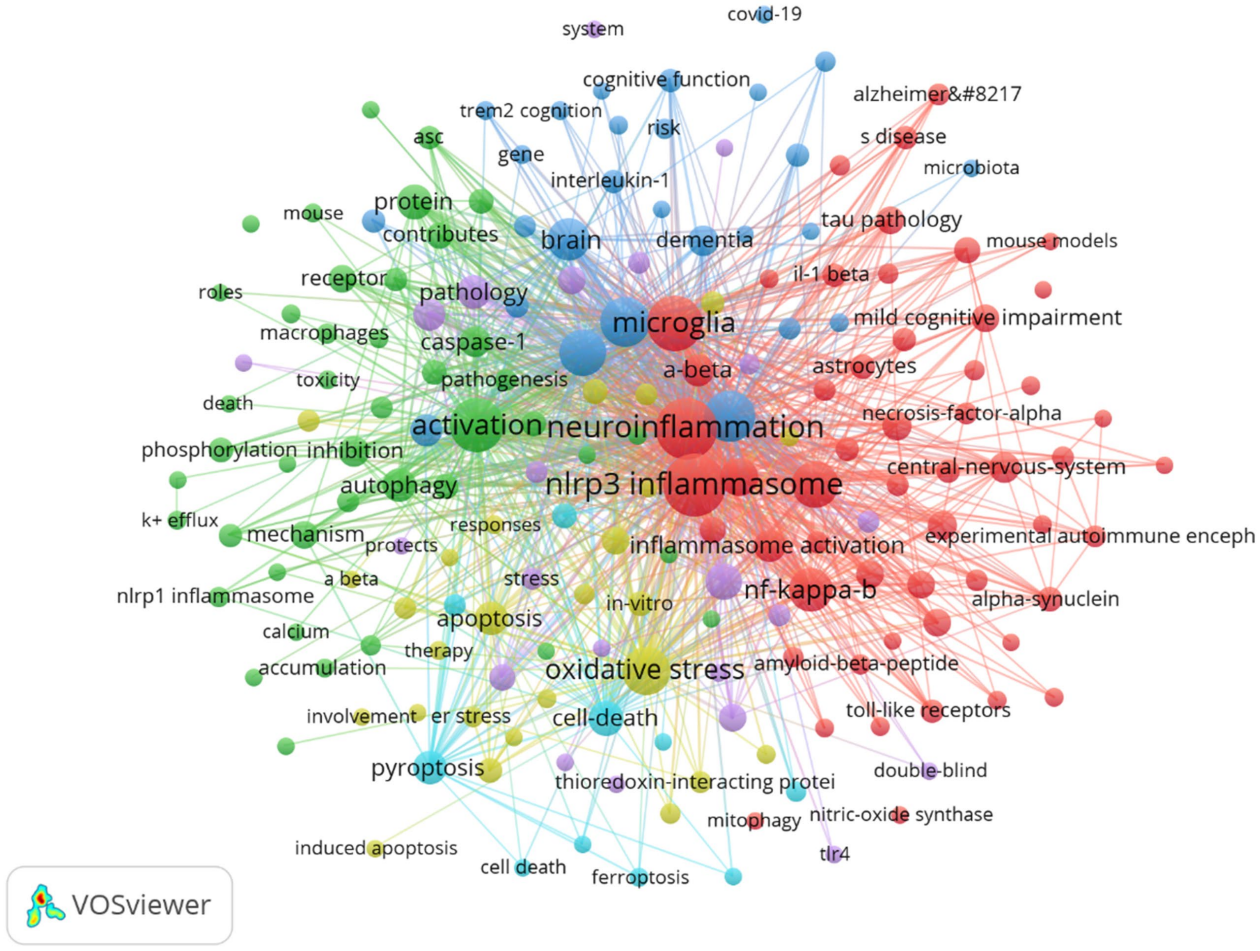
Network map of high-frequency keywords.

**Figure 16:**
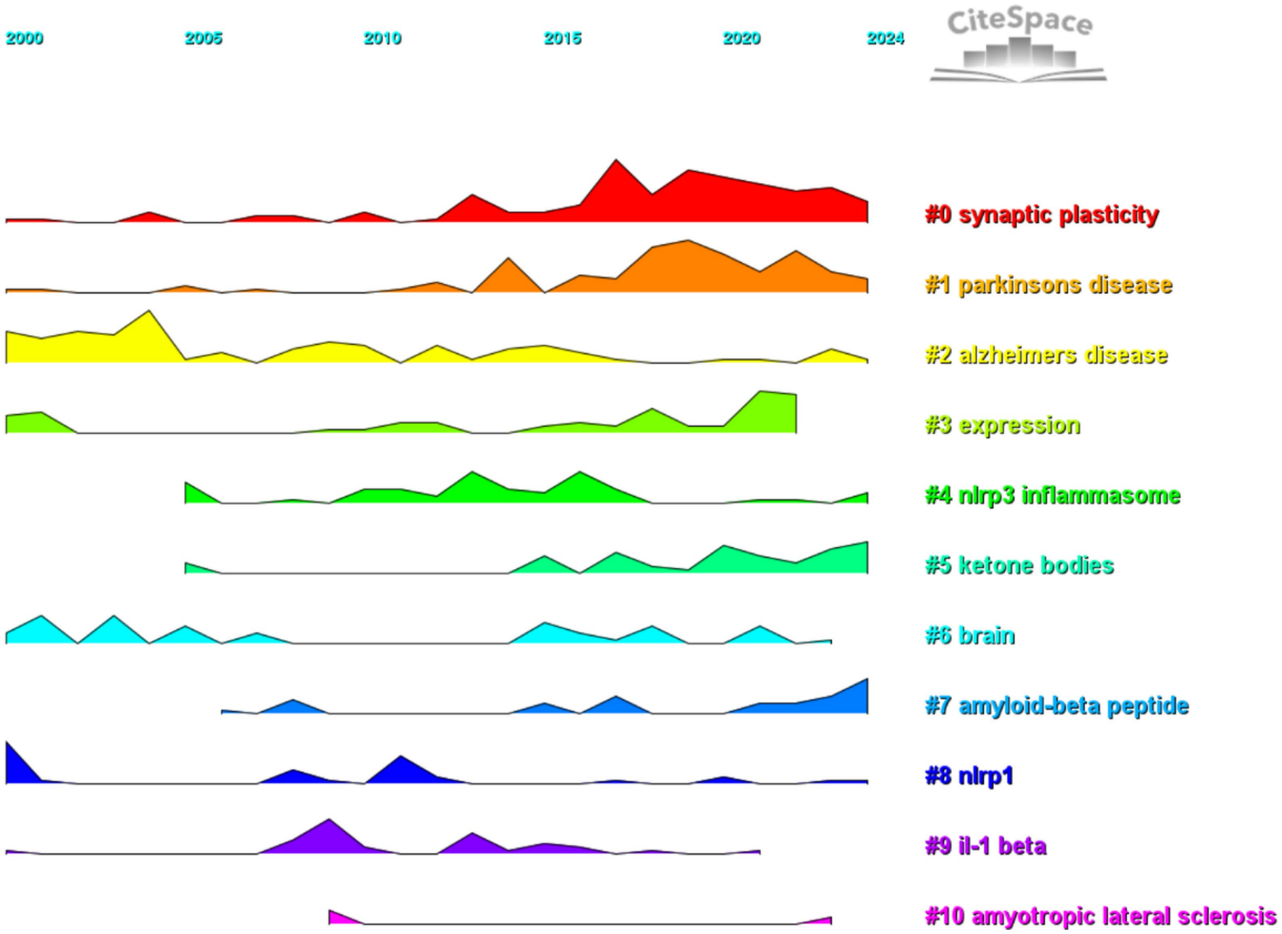
Bursting map of cited literature.

By analyzing the keywords, we can quickly gain an understanding of a field and its direction. According to the co-occurrence of keywords in VOSviewer, the most popular keyword was nlrp3 inflammasome (409), followed by neuroinflammation (377), microglia (283), activation (281), and amyloid-beta (231) (Table 7, Figure 15, and Supplementary Figure S1). We removed the useless keywords and constructed a network containing 182 keywords with at least 11 occurrences, yielding a total of 5 different clusters. Cluster 1 (red) contains 54 keywords, including neuroinflammation, nlrp3 inflammasome, microglia, mouse model, nf kappa b, innate immunity, aging, astrocyte, blood–brain barrier, cognitive deficits, cytokine, genome-wide association, mitophagy, multiple sclerosis, nitric oxide, t cell, tau pathology, toll-like receptor. Cluster 2 (green) has 43 keywords, including activation, autophagy dysfunction, inhibition, interleukin-1 beta, mechanism, phagocytosis, toxicity, macrophage, pathway, accumulation, clearance, dysfunction, caspases −1, hippocampus, injury, oligomers, peptide, protein. Cluster 3 contains 27 keywords (blue), including amyloid beta, inflammasome, inflammation, brain dementia, apoe, cognitive decline, COVID-19, gut microbiota, infection, insulin resistance, metabolism, risk, term2. Cluster 4 contains 26 keywords (yellow), including oxidative stress, apoptosis, mitochondria, neurotoxicity, therapy, responses, er stress, involvement, degradation, stroke, memory deficits, neuroprotection, therapy, transgenic mice. Cluster 5 contains 22 keywords (purple), including cognitive impairment, deficits, depression, double-blind, impairment, model, nrf2, pathway, protect, rat model, signaling pathway, stress. Cluster 6 contains 10 keywords (sky blue), including amyloid beta, cell death, ferroptosis, gasdermin d, inhibitor, necroptosis, pyroptosis. We created a volcano plot with CiteSpace to visualize the changes in research hotspots over time (Figure 16 and Supplementary Figure S2), and we found that [fill in the blank with the specific research hotspots you want to mention] are the current research hotspots. (You need to provide the specific content for the blank part according to your actual situation.)

**Table 7:** High frequency keyword table.

Rank	Keyword	Counts	Rank	Keyword	Counts
1	nlrp3 inflammasome	409	11	Brain	131
2	Neuroinflammation	377	12	Neurodegeneration	98
3	Microglia	283	13	Cognitive impairment	91
4	Activation	281	14	Cell-death	81
5	Amyloid-beta	231	15	Protein	79
6	Inflammasome	221	16	Pathology	73
7	Oxidative stress	200	17	Autophagy	73
8	Mouse model	182	18	Apoptosis	72
9	Inflammation	180	19	Pyroptosis	70
10	nf-kappa-b	154	20	Model	62

Using CiteSpace, we identified 50 of the most influential citation bursts in the field of inflammasome research related to Alzheimer’s disease. The most highly cited reference is “The NLRP3 and NLRP1 inflammasomes are activated in Alzheimer’s disease,” published in Molecular Neurodegeneration with Marina Saresella as the first author. This study demonstrates that the mRNAs of inflammasome components (NLRP1, NLRP3, PYCARD, caspase 1, 5, and 8) and downstream effectors (IL-1β, IL-18) are upregulated in both severe and mild AD. In severe AD, there is a significant increase in the co-expression of NLRP3 with caspase 1 or caspase 8 in monocytes. Additionally, monocytes co-expressing NLRP1 and NLRP3 with PYCARD are elevated in both severe and moderate AD. Confocal microscopy confirmed the activation of NLRP1 and NLRP3 inflammasomes in AD, with a notable increase in the production of pro-inflammatory cytokines IL-1β and IL-18 by monocytes. In individuals with mild cognitive impairment (MCI), NLRP3 expression was elevated, but PYCARD or caspase 1 was not, indicating that functional inflammasomes were not assembled. This was confirmed by the lack of co-localization and cytokine production. Strategies targeting inflammasome activation may offer therapeutic potential for AD (12). All 50 references were published between 2000 and 2024, indicating their sustained citation over the past two decades. Notably, five of these papers are currently at the peak of citation, suggesting that research on inflammasomes in Alzheimer’s disease will continue to garner significant attention.

## 4 Discussion

In this study, we employed bibliometric methods to elucidate the knowledge base and emerging trends of inflammasomes in Alzheimer’s disease (AD) neuroinflammation, aiming to inform readers of the latest insights and future research directions. Additionally, we introduced key journals, institutions, and authors that readers can consult to gain further understanding of this topic.

### 4.1 General trends

The concept of the inflammasome was first introduced in 2002. As research has progressed, the number of publications has increased rapidly since 2013, with further acceleration in 2020. This trend indicates that the interaction between inflammasomes and the pathogenesis of AD is gaining significant attention and importance in AD research. China, the United States, Italy, the United Kingdom, and Germany have published numerous articles in this field. Publications from China account for 41.13% of the total, with high citation frequency and a centrality of 0.13. Among the top 10 institutions by publication volume, four are from China, three from Germany, two from the United Kingdom, and one from the United States, highlighting China as a leading country in this field. Papers from the United States have been cited 24,534 times (Table 1), with a citation/publication ratio of 92.23, ranking third globally, suggesting high quality. Notably, China and the United States together account for 64.71% of total publications, underscoring their leading positions in inflammasome research in Alzheimer’s disease. Figure 5 illustrates the cooperation network. The United States collaborates closely with the United Kingdom, Italy, and Germany, while China collaborates more with Japan, South Korea, and India. Further analysis shows that institutions tend to collaborate within their own countries. We advocate for enhanced international cooperation to overcome academic barriers.

As shown in Tables 3, 4, the *International Journal of Molecular Sciences* (50 articles, 4.43%) publishes the most papers on inflammasomes in Alzheimer’s disease. Among the top 10 journals by publication volume, two also rank among the top 10 co-cited journals (*Journal of Neuroinflammation*, *Journal of Alzheimer’s Disease*), indicating their significant influence in this field. Notably, all top 10 journals are ranked in Q1 or Q2, suggesting that the quality of publications in this field is generally high.

According to Table 5, Heneka, M.T. has the most papers (18, 1.60%) and the highest number of citations (600), indicating his significant influence and outstanding contribution to the field of inflammasomes in AD. Heneka, M.T. is a professor at the University of Bonn, specializing in neurodegenerative diseases and molecular biology. In 2013, Heneka, M.T. et al. published a paper titled “NLRP3 inflammasome is activated in Alzheimer’s disease and contributes to pathology in APP/PS1 mice” in *Nature*, confirming the association between NLRP3 and AD pathogenesis. This article ranks sixth (98 citations) among the top 10 most co-cited references and exhibits the strongest citation burst (52.54) (13).

### 4.2 Research hotspots

Among the top 10 co-cited references, seven pertain to the NLRP3 inflammasome (10–16), indicating it is the most extensively studied. Its activation and pathological responses play a crucial role in neuroinflammatory processes. Four studies explore the relationship between the NLRP3 inflammasome and the pathological deposition of amyloid-beta and tau proteins, noting that it can be activated by these proteins and accelerate their accumulation (10, 11, 14, 17). Two articles examine the role of other inflammasomes in Alzheimer’s disease and neurodegenerative disorders. Two articles discuss additionally microglial activation in the progression of AD (12, 18). According to, five articles are at their citation peak, with four related to NLRP3 inflammasome activation and pathways, suggesting that these mechanisms will remain a focal point in Alzheimer’s research. The other two articles focus on microglia, highlighting their significant research potential as primary immune cells in neuroinflammatory responses.

Keywords reflect research hotspots and directions in a specific field. According to Table 7 the most frequent keyword is “NLRP3 inflammasome” (409) followed by “neuroinflammation” (377) “microglia” (283)"activation” (281) and “amyloid-beta” (231). These keywords indicate that the NLRP3 inflammasome is the most studied neuroinflammatory component closely related to Alzheimer’s disease (AD) pathogenesis. In neuroinflammatory responses microglial activation is the initial phase. Amyloid-beta is a pathological product of AD with pro-inflammatory effects during deposition inducing inflammasome activation and assembly.

Cluster analysis of the keywords reveals that the first group relates to innate immunity and inflammatory signaling pathways. The second group pertains to autophagy, with dysfunction potentially leading to AD progression. The third group involves causes of neuroinflammation, including infection, COVID-19, and insulin resistance. The fourth group is associated with oxidative stress, a major contributor to AD. The fifth group pertains to mouse experiments. The sixth group is connected to pyroptosis, a form of cell death induced by inflammatory cytokines released by inflammasomes.

## 5 Limitations

Firstly, this study is a bibliometric analysis; CiteSpace and VOSviewer cannot fully replace systematic searches. Secondly, all data were retrieved from the WoSCC database, which may exclude some papers not included in it. However, WoSCC is the most commonly used database in scientometric analyses, and its data can represent a significant portion of information. Visualization analysis based on reference data aids researchers in intuitively understanding research hotspots, evolutionary processes, and development trends of inflammasomes in Alzheimer’s disease.

## 6 Conclusion

In conclusion, the inflammasome is an important mechanism in the pathogenesis of AD, with the NLRP3 inflammasome being the best known. Unfortunately, between 2000 and 2024, the number of relevant literature peaks in 2023 and shows a decreasing trend in 2024, suggesting that this research hotspot may be fading. For now, the research hotspot in this area appears to be declining. In the case of Alzheimer’s disease, there is an urgent need to explore new pathogenic mechanisms. This is undoubtedly an important direction that requires in-depth thinking and breakthroughs, as new drugs targeting Aβ and Tau proteins have not yet been introduced, nor have there been significant advances in treatment.

## Data Availability

The raw data supporting the conclusions of this article will be made available by the authors, without undue reservation.

## Supplementary material

Supplementary Figure S1 Density map of keywords.

Supplementary Figure S2 Clustering map of keywords.
