# Supplementary figures and images for: Tracing inflammasomes in Alzheimer’s: insights from bibliometric analysis
Source: Front Neurol. 2025 Jun 5;16:1540083. doi: 10.3389/fneur.2025.1540083 (PMC12176572; doi:10.3389/fneur.2025.1540083)

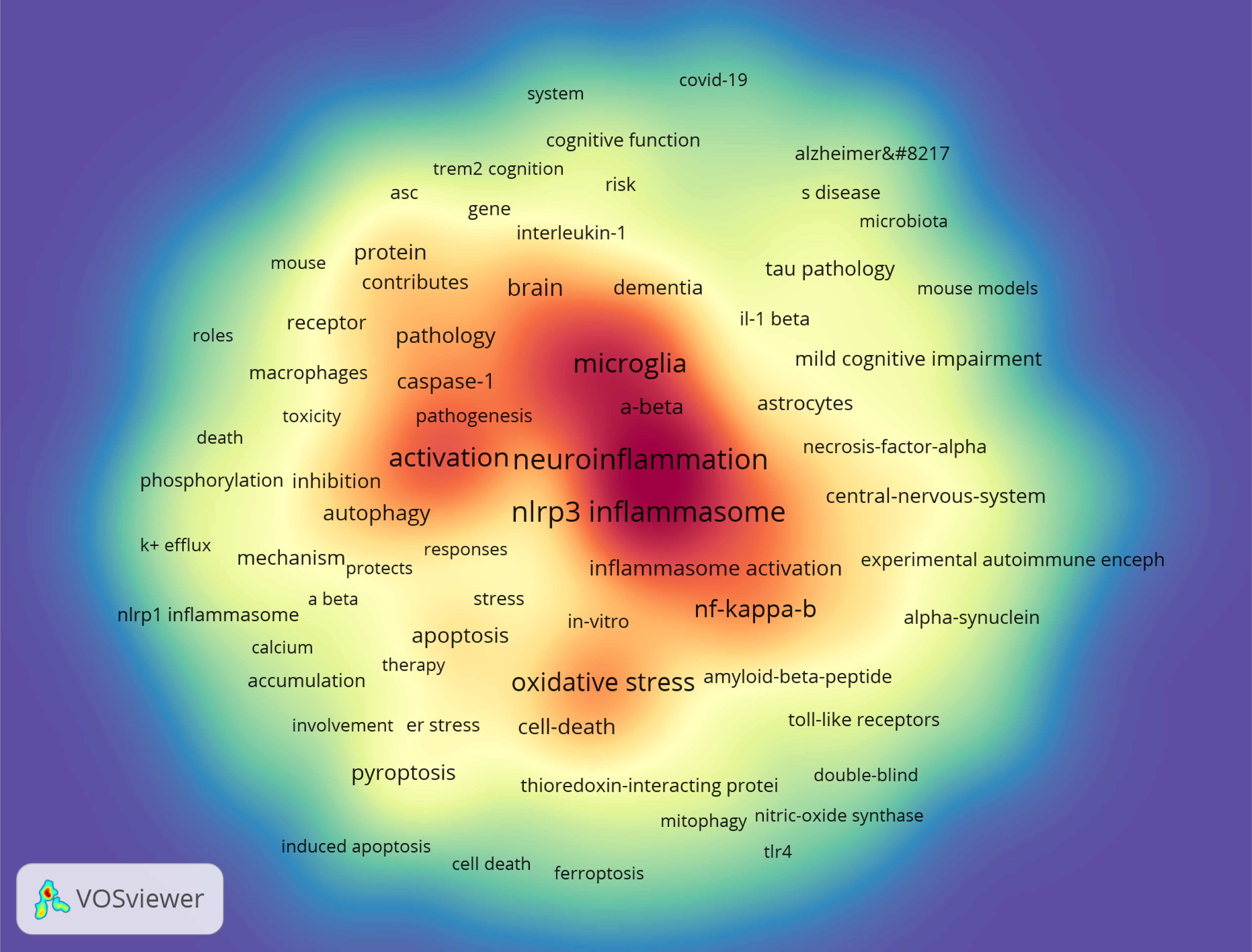

Supplement: Supplementary Figure S1 — Density map of keywords. [file Image_1.tif]

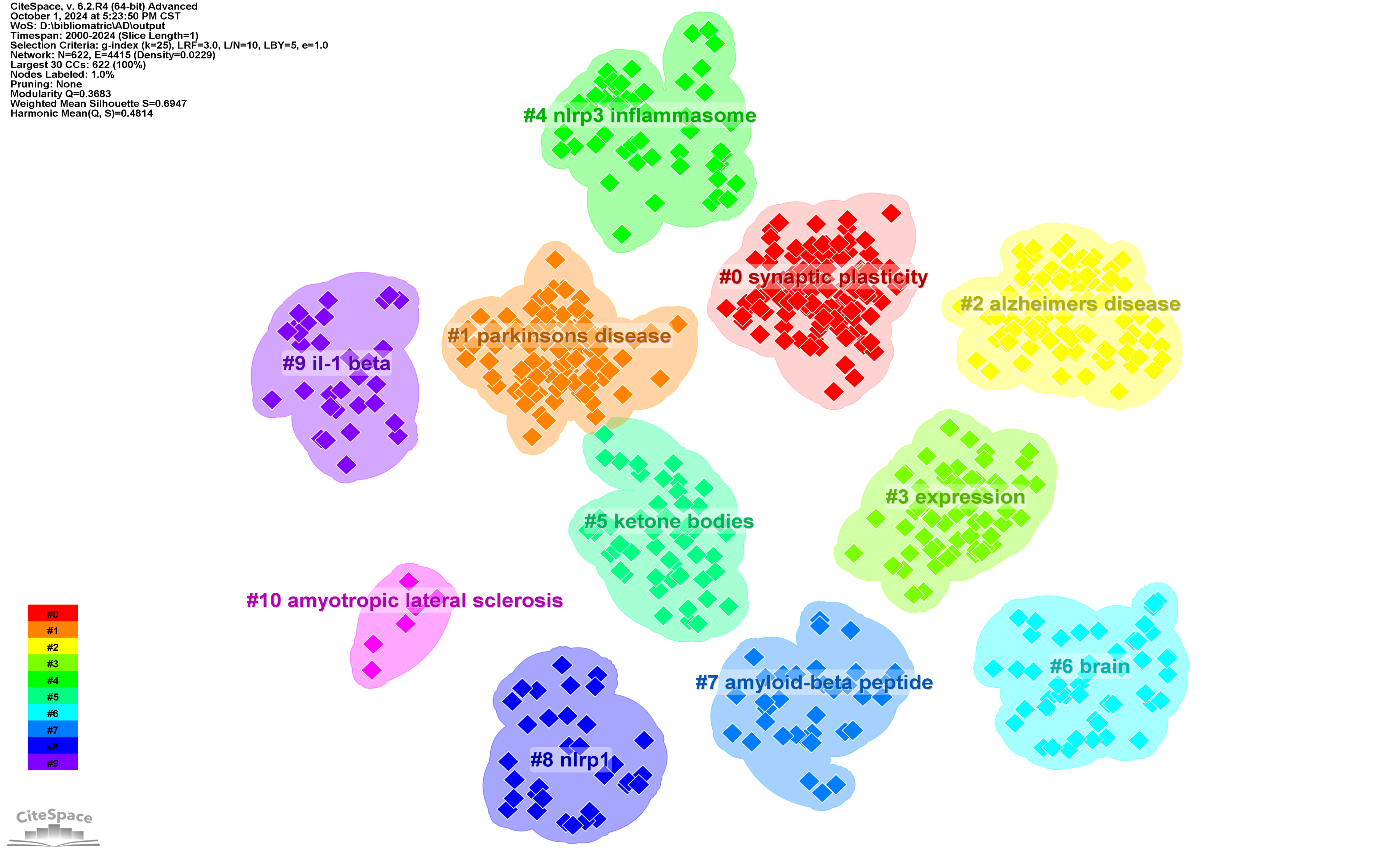

Supplement: Supplementary Figure S2 — Clustering map of keywords. [file Image_2.tif]
